# ﻿Comparative cytogenetic patterns in Carangidae fishes in association with their distribution range

**DOI:** 10.3897/CompCytogen.v15.i4.69638

**Published:** 2021-12-01

**Authors:** Rodrigo Xavier Soares, Clóvis Coutinho da Motta-Neto, Gideão Wagner Werneck Félix da Costa, Marcelo de Bello Cioffi, Luiz Antônio Carlos Bertollo, Amanda Torres Borges, Wagner Franco Molina

**Affiliations:** 1 Departament of Cell Biology and Genetics, Biosciences Center, Universidade Federal do Rio Grande do Norte, Natal, RN, 59078970, Brazil; 2 Fish Cytogenetics Laboratory, Departament of Genetics and Evolution, Universidade Federal de São Carlos, São Carlos, SP, C.P. 676, Brazils

**Keywords:** Conservative karyotype, Fish cytogenetics, karyotype evolution, pelagic fishes

## Abstract

Carangidae are an important and widespreaded family of pelagic predatory fishes that inhabit reef regions or open ocean areas, some species occupying a vast circumglobal distribution. Cytogenetic comparisons among representatives of its different tribes help to understand the process of karyotype divergence in marine ecosystems due to the variable migratory ability of species. In this sense, conventional cytogenetic investigations (Giemsa staining, Ag-NORs, and C-banding), GC base-specific fluorochrome staining and FISH mapping of ribosomal DNAs were performed. Four species, *Elagatisbipinnulata* (Quoy et Gaimard, 1825) and *Seriolarivoliana* (Valenciennes, 1883) (Naucratini), with circumtropical distributions, *Gnathanodonspeciosus* (Forsskål, 1775) (Carangini), widely distributed in the tropical and subtropical waters of the Indian and Pacific oceans, and *Trachinotuscarolinus* (Linnaeus, 1766) (Trachinotini), distributed along the western Atlantic Ocean, were analyzed, thus encompassing representatives of three out its four tribes. All species have diploid chromosome number 2n = 48, with karyotypes composed mainly by acrocentric chromosomes (NF = 50–56). The 18S rDNA/Ag-NORs/GC+ and 5S rDNA loci were located on chromosomes likely homeologs. Karyotypes showed a pattern considered basal for the family or with small variations in their structures, apparently due to pericentric inversions. The migratory capacity of large pelagic swimmers, in large distribution areas, likely restricts the fixation of chromosome changes in Carangidae responsible for a low level of karyotype diversification.

## ﻿Introduction

The spatial distribution of biodiversity is related to the existing or past physical and environmental conditions. In this context, fishes provide good models for investigating the association between chromosome diversity and environmental characteristics of different regions and ecosystems (Molina et al. 2014). Some fish groups show a high congruence between their high biodiversity and patterns of karyotype diversifications (Bertollo et al. 2000). Among freshwater fishes, the species diversity is linked to an evident allopatric isolation scenarios leading to the fixation of chromosome rearrangements (Moreira-Filho and Bertollo 1991; Costa et al. 2019). In marine fishes, in addition to environmental physical barriers, the karyotype diversification is also associated with the limited dispersion or colonization capacity of the species (Molina and Galetti Jr 2004; Molina et al. 2014), together with the effective size of the populations (Riginos et al. 2016; Motta-Neto et al. 2019).

The marine environment is both extensive and multidimensional due to its varied ecological patterns, thus providing complex evolutionary conditions that impacts the genetic structure of species (Rocha 2003). Egg types and length of the larval period are not the only biological factors predicting the geographic structure of the reef fish populations (Shulman and Bermingham 1995). Larval behavior and habitat availability are equally important ones for maintaining the population structure (Kohn and Clements 2011). In demersal species, for example, the association of biogeographic barriers physical factors such as currents, and the dispersion of eggs and larvae, can promote larval retention and the maintenance of genetically connected populations over long distances (Taylor and Hellberg 2003; Craig et al. 2007; Saenz‐Agudelo et al. 2011). The maintenance of widely distributed populations is particularly more limited in reef species, as exemplified by the absence of circumglobal distribution in any Gobiidae species, the most species-rich marine group (Gaither et al. 2016). On the other hand, in groups with pelagic habits and vast oceanic distributions, genetic patterns are established by migrating adults, larval behavior, and dispersal under limits of physical or ecological barriers (Palumbi 1994).

The investigation of environmental effects on the genetic diversity of marine fish species depends on favorable spatial models, which have been used to identify causes and factors that promote their karyotype differentiation (Accioly et al. 2012; Molina et al. 2012; Amorim et al. 2017; Motta-Neto et al. 2019). Additionally, it is also advantageous to add an integrated view of contemporary ecological and environmental patterns associated with the historical biogeography of the groups (Molina 2007; Molina et al. 2014; Amorim et al. 2017).

A negative correlation was found when associating the dispersive potential of the pelagic larvae with the karyotype diversification in reef fish (Molina and Galetti Jr 2004; Sena and Molina 2007). Although an increasing number of large pelagic species have recently been the target of more detailed cytogenetic analyses (Soares et al. 2013, 2014, 2017), studies on the dispersive potential and chromosome diversification have been neglected in pelagic fish. It is estimated that almost three hundred of marine fish species have a circumtropical distribution (Gaither et al. 2016), and, among them, the Carangidae family (Carangoidei, Carangiformes) stands out with several species reaching wide oceanic distributions (Froese and Pauly 2020).

Carangidae are pelagic fishes with high swimming capacity, composed of 31 genera and 150 presently recognized species (Nelson et al. 2016; Fricke et al. 2020), with very variable hydrodynamic body adaptations, ranging from slender to deep-bodied ones (Honebrink 2000). Their phylogenetic relationships based on morphological (Smith‐Vaniz 1984) and molecular data (Damerau et al. 2017) point out four monophyletic tribes, namely Naucratinae, Scomberoidinae, Caranginae, and Trachinotinae. The ancient origin of this group and its radiation during the Cretaceous period offer extensive spatial scenarios (Honebrink 2000) for analyzing its genetic diversity (Santini and Carnevale 2015). Thus, cytogenetic analyses in groups with such a wide geographic distribution, provide favorable tools for understanding the role of biogeographic barriers and the dispersive potential on karyotype changes.

In view of the environmental complexity to which the marine fish groups are subjected, the investigation of their chromosome change patterns must consider wide taxonomic, biogeographic, and different biological models. In the context of the marine environment, cytogenetic analyses in groups with a wide geographic distribution, such as Carangidae, provide an understanding of the role of biogeographic barriers and the dispersive potential on karyotype changes. Therefore, we performed cytogenetic analyses using conventional and molecular protocols in *Elagatisbipinnulata* (Quoy et Gaimard, 1825) (Rainbow runner), *Seriolarivoliana* (Valenciennes, 1883) (Greater amberjack), *Gnathanodonspeciosus* (Forsskål, 1775) (Golden trevally), and *Trachinotuscarolinus* (Linnaeus, 1766) (Florida pompano), which have extensive geographical distribution. The patterns of their karyotype evolution were discussed in relation with the dispersive potential estimated by their geographic distribution.

## ﻿Material and methods

### ﻿Individuals and mitotic chromosome preparation

Cytogenetic analyses were performed on the species *Elagatisbipinnulata* (n = 15; 10 males; 5 females), and *Seriolarivoliana* (n = 4; 1 male; 3 females), both from off the São Pedro and São Paulo archipelago (00°56'N, 29°22'W) located in the Meso-Atlantic region; in *Gnathanodonspeciosus* (n = 2; juveniles), from the Pacific and Indo-Pacific, obtained through ornamental fish traders, and *Trachinotuscarolinus* (n = 10; 4 males; 6 females), from cultivated stock of the coast of Florida (USA). The samples were collected with the authorization of the Brazilian environmental agency ICMBio/ SISBIO (License #19135-4, #131360-1 and #27027-2).

The individuals were subjected to in vivo mitotic stimulation according to Molina et al. (2010). Chromosome preparations were obtained from short-term culture (Gold et al. 1990) using tissue suspensions from the anterior portion of the kidney. All the experiments followed ethical protocols and anesthesia conducted with clove oil prior the animals were sacrified. The process was approved by the Animal Ethics Committee of Federal University of Rio Grande do Norte (Protocol 44/15).

### ﻿Standard cytogenetic procedures

The nucleolus organizing regions (**NORs**) and the chromosomal heterochromatin content were analyzed according to the C-banding and Ag-NOR methods, reported by Sumner (1972) and Howell and Black (1980), respectively. The CG rich sites were vizualized with the base-specific mithramycin (**MM**) fluorochrome and DAPI staining (Schweizer 1976).

### ﻿Fluorescence in situ hybridization (FISH)

FISH was performed according to Pinkel et al. (1986). The 18S and 5S rDNA probes were obtained using PCR with DNA template from *Rachycentroncanadum* (Linnaeus, 1766) (Euteleostei, Rachycentridae) and primer pairs NS1 5'-GTA GTC ATA TGC TTG TCT C-3' and NS8 5'-TCC GCA GGT TCA CCT ACG GA-3' (White et al., 1990) and A 5'-TAC GCC CGA TCT CGT CCG ATC-3' and B 5'-CAG GCT GGT ATG GCC GTA AGC-3' (Pendás et al. 1994), respectively. The probes were labeled by nick translation (Invitrogen, Thermo Fisher Scientific, Waltham, MA, USA) with digoxigenin-11-dUTP and biotin-14-dATP for the 18S rDNA, and 5S rDNA, respectively. Hybridization signals were detected using anti-digoxygenin-rhodamine (Roche, Mannheim, Alemanha) and streptavidin-FITC (Vector Laboratories, Burlingame, CA, USA), for the 18S and 5S rDNA probes, respectively, according to the manufacturer’s specifications. The chromosomes were counterstained with Vectashield/DAPI (Vector Laboratories, Burlingame, CA, USA).

### ﻿Cytogenetic analyses

At least 30 metaphase spreads per individual were analyzed to confirm the chromosome number, karyotype structure, and FISH results. Images were photographed using an Olympus BX51 epifluorescence microscope coupled to an Olympus DP73 digital image capture system (Olympus Corporation, Ishikawa, Japan) with the cellSens (Version 1.9 Digital, Tokyo, Kanto, Japan) software. Chromosomes were classified as metacentric (m), submetacentric (sm), subtelocentric (st), and acrocentric (a) according to their arm ratios (Levan et al. 1964). To count the chromosome arms (NF), the m, sm, and st chromosomes were considered with two arms and the acrocentric chromosomes (or if classified indistinctly as st/a) with only one arm.

### ﻿Estimation of maximum linear geographic distribution and total area occupied by species

The maximum linear geographic distribution distance (MLD) and the occupied area by each species (OA) were obtained through the Ocean Biogeographic Information System (Obis 2020). The OBIS platform performs analyses using an online management software that accesses spatial distribution databases associated with the highest taxonomic levels, geographic area, time, and depth, providing a map of localities related to environmental data. Based on the maps for each species, the corresponding files were loaded in the public domain software Image J (Rasband 2018), allowing us to measure the distribution areas and the linear distribution axis distances of each species and tribe, as presented in Table 1. To calculate the proportionality of the distances and distribution areas, the data for each species and each tribe were defined proportionally to the data of *Elagatisbipinnulata*, a species chosen as a representative parameter of the maximum circumglobal distribution for the family.

### ﻿Statistical analysis

The descriptive statistical analysis (Table 1) and Spearman’s rank correlation coefficient were calculated using the RStudio software. The Shapiro-Wilk test was used to normality afferition. These tests were conducted to determine the correlation between the number of chromosome arms (NF) and the measure of the species’ geographic distribution, as indicated by the maximum axis of linear geographic distribution (MLD) and total area occupied (OA). The level of significance adopted was *p* < 0.05. The cytogenetic database for the Carangidae species was obtained from an exhaustive up-to-date online review. The cytogenetic data (2n, karyotype composition, NF) covered the studies published among the years 1974 and 2021, and was used in comparative intra- and inter-group analyses. The search was developed in representative research portals, encompassing Google Scholar (https://scholar.google.com.br/), SciELO (http://www.scielo.br/), Portal de Periodicos (http://www.periodicos.capes.gov.br/), Web of Science ResearchGate (http://www.researchgate.net), and included the extensive review by Arai (2011).

**Table 1.** Cytogenetic data from species of the family Carangidae and their maximum linear geographic distribution (MLD) and occupied area (OA) and ratio with the maximum distribution values defined for the family. Vertical bars represent the set of parameters available to the species.

Species	MLD Km × 10^4^	% LGDmax	OA Km^2^ × 10^4^	% OAmax	2n	Karyotype	NF	Ref.
**Naucratini**								
*Elagatisbipinnulata* (Quoy et Gaimard, 1825)	3.80	100	977.05	100	48	2st+46a	50	1
*Seriolinanigrofasciata* (Rüppell, 1829)	1.34	40	213.16	30	48	48a	48	2
*Seriolarivoliana* Valenciennes, 1833	3.14	90	446.50	50	48	2sm+2st+44a	52	1
*Serioladumerili* (Risso, 1810)	2.05	60	526.26	60	48	2sm+46a	50	3
					48	2sm+2st+44a	52	4
*Seriolaquinqueradiata* Temminck et Schlegel, 1845	0.13	10	18.54	20	48	2sm+2st+44a	52	5
*Seriolalalandi* Valenciennes, 1833	1.43	40	454.94	50	48	2m+2sm+6st+38a	58	6
***Average values***	1.98	**60**	439.41	**50**			**51.7**	
**Scomberoidini**								
*Scomberoideslysan* (Forsskål, 1775)	2.15	60	523.60	60	48	4m/sm+44a	52	7
*Oligoplitessaliens* (Bloch, 1793)	0.60	20	36.95	10	48	4m/sm+44st/a	52	8
***Average values***	1.37	**40**	280.28	**30**			**52**	
**Carangini**								
*Alectisciliaris* (Bloch, 1787)	2.75	80	579.72	60	48	48a	48	9
*Alepesdjedaba* (Forsskål, 1775)	1.33	40	161.50	20	56	56a	56	10
*Alepesmelanoptera* (Swainson, 1839)	0.83	30	122.57	20	48	2sm+46a	50	10
*Atropusatropos* (Bloch et Schneider, 1801)	0.71	20	43.83	10	48	48a	48	7
*Atulemate* (Cuvier, 1833)	1.48	40	483.84	50	50	14sm+36a	64	11
*Carangoidesarmatus* (Rüppell, 1830)	0.99	30	197.62	30	48	2st+46a	50	7
*Carangoidesequula* (Temminck et Schlegel, 1844)	1.48	40	221.06	30	48	2st+46a	50	9
*Carangoidesbartholomaei* (Cuvier, 1833)	0.73	20	219.65	30	48	6sm+42a	54	12
*Caranxpraeustus* Anonymous (Bennett), 1830	0.67	20	41.97	10	48	10m/sm+28a	58	7
*Caranxlatus* Agassiz, 1831	1.14	40	212.55	30	48	2sm+46a	50	12
*Caranxlugubris* Poey, 1860	3.59	100	379.16	40	48	2sm+46a	50	13
*Caranxignobilis* (Forsskål, 1775)	2.21	60	590.42	70	48	2sm+46a	50	14
*Caranxsexfasciatus* Quoy et Gaimard, 1825	2.48	70	875.01	90	48	2st+46a	50	9
*Chloroscombruschrysurus* (Linnaeus, 1766)	1.33	40	422.56	50	48	48a	48	15
*Gnathanodonspeciosus* (Forsskål, 1775)	2.47	70	615.10	70	48	2st+46a	50	1
*Megalaspiscordyla* (Linnaeus, 1758)	1.06	30	336.44	40	50	2st+48a	50	10
*Selenesetapinnis* (Mitchill, 1815)	1.23	40	298.39	40	46	2sm+44a/2m+44a	48	16
*Selenevomer* (Linnaeus, 1758)	0.69	20	289.54	30	48	2st+46a	50	16
*Selenebrownii* (Cuvier, 1816)	0.40	20	46.20	10	48	48a	48	16
*Trachurusjaponicus* (Temminck et Schlegel, 1844)	0.23	10	40.34	10	48	4m+14sm+12st+18a	78	9
*T.mediterraneus* (Steindachner, 1868)	0.58	20	122.38	20	48	4m+6sm+38st/a	58	17
	0.69				48	4m+4sm+14st+26a	70	18
*T.trachurus* (Linnaeus, 1758)	1.32	20	423.31	50	48	2sm+46a	50	18
***Average values***	2.75	**40**	327.54	**40**			**53.4**	
**Trachinotini**								
*Trachinotusgoodei* Jordan et Evermann, 1896	0.67	20	89.73	10	48	4m/sm+44a	52	19
*T.carolinus* (Linnaeus, 1766)	0.69 0.19	20 10	122.06	20	48	8m/sm+40a	56	19
			25.22	10	48	4m+4sm+40a	56	1
*T.falcatus* (Linnaeus, 1758)	1.30	40	265.56	30	48	10m/sm+38a	58	19
			125.64	20	48	2m+2st+44a	52	20
*T.ovatus* (Linnaeus, 1758)	0.67	20	89.73	10	48	2m+4sm+42st/a	54	10
***Average values***	0.69	**20**	122.06	**20**			**54.4**	

**Notes**: 1 – present study; 2 – Tripathy and Das (1988); 3 – Vitturi et al., (1986); 4 – Sola et al. (1997); 5 – Ida et al. (1978); 6 – Chai et al. (2009); 7 – Das et al. (1980); 8 – Castro-Leal et al. (1998); 9 – Murofushi and Yosida (1979); 10 – Choudhury et al. (1993); 11 – Lee and Loo (1975); 12 – Jacobina et al. (2014a); 13 – Jacobina et al. (2014b); 14 – Patro and Prasad (1979); 15 – Accioly et al. (2012); 16 – Jacobina et al. (2013); 17 – Vasiliev (1978); 18 – Caputo et al. (1996); 19 – Jacobina et al. (2012); 20 – Nirchio et al. 2014.

## ﻿Results

### ﻿Cytogenetic data

All species had 2n = 48, but with different karyotypes. While *E.bipinnulata* and *G.speciosus* shared karyotypes with 2st+46a (NF = 50), *S.rivoliana* has 2sm+2st+44a (NF = 52), and *T.carolinus* has 4m+4sm+40a (NF = 56). No evidence of the presence of differentiated sex chromosomes was found.

**Figure 1.**
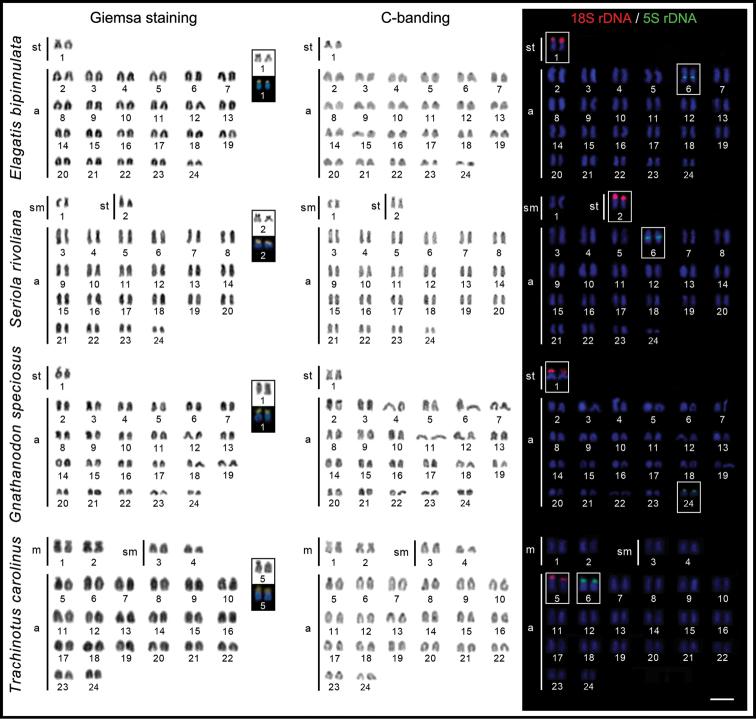
Karyotypes of *Elagatisbipinnulata*, *Seriolarivoliana*, *Gnathanodonspeciosus*, and *Trachinotuscarolinus* arranged after Giemsa staining (Ag-NORs and MM^+^/DAPI- sites, highlighted), C- banding, and double-FISH with 18S rDNA (red) and 5S rDNA (green) probes. The chromosome pairs were tentatively numbered. Scale bar: 5 μm.

C-positive heterochromatic blocks were located mainly in the pericentromeric regions and in the terminal regions of some chromosome pairs to a lesser extent (Fig. 1). An unique Ag-NOR site, coincident with conspicuous heterochromatic and MM^+^/DAPI- regions was found in the four species. In *E.bipinnulata* and *G.speciosus*, these regions were located in the end of the short arms of the large st pair No. 1. In *S.rivoliana* and *T.carolinus*, they were located in the same position, but in the similarly sized subtelocentric pair No. 2 in *S.rivoliana*, and in a large a element marked as pair No. 5 in *T.carolinus* (Fig. 1). In all species, the Ag-NOR sites corresponded to the positive 18S rDNA hybridization signals.

The 5S rDNA loci were also unique, but with an interstitial or terminal distribution in a pairs of similar size among the species, and non-syntenic with the 18S ones. In *E.bipinnulata* and *S.rivoliana*, they were interstitially located in the q arms of the pair labelled as No. 6; in the terminal region of the short arms of the pair labelled as No. 6 in *T.carolinus*, and in the pericentromeric region of the smallest chromosome pair No. 24 in *G.speciosus* (Fig. 1).

### ﻿NF average and geographic distribution

The average number of the chromosome arms (NF average) showed an negative correlation with the averages of linear distances of distribution and areas occupied for each tribe. In fact, the NF average showed be progressively divergent on the NF considered as basal for the family (NF = 50) in Naucratini (51.7), that encompass an average linear distribution distance equivalent to 60% of the greatest distance established for the family (LD), and 50% concerning the largest occupied area (LOA); Scomberoidini (52), with 40% (LD) and 30% (LOA), Carangini (53.4), with 40% (LD) and 40% (LOA) and Trachinotini (54.4), with 20% (LD) and 20% (LOA) (Table 1).

### ﻿Statistical data

The average of the two distribution measures (MLD and OA) and NF values showed evidences on an statistically supported relationship between karyotype and geographic distribution, that encompass a synergic set of ecological, adaptive, and migratory characteristics. The variables MLD (p = 0.001), OA (*p* = 0.001), and NF (*p* < 3.097e-07) did not present a normal distribution (Shapiro-Wilk test). The analysis revealed a high correlation between the MLD and OA (Pearson’s correlation r = 0.829, *p* ≤ 0.05). The NF values showed a moderate negative Pearson’s correlation coefficient with the MLD (r = -0.419, *p* = 0.0144) and modest negative correlation with OA (r = -0.876, *p* = 0.043).

## ﻿Discussion

In contrast to other marine fish groups, Carangidae have a representative set of cytogenetic data (Table 1), now including new data for species of the genera *Elagatis* Bennett, 1840, *Seriola* Cuvier, 1816, *Gnathanodon* Bleeker, 1850, and *Trachinotus* Lacepède, 1801, reaching 22% of its species, encompassing all tribes. This repertoire shows remarkable conservation of the diploid number, with 2n = 48 occuring in 88% of the species. On the other hand, divergences exits in the karyotype compositions, with variation in chromosome arms (NF) from 48 to 78 (Table 1). Karyotypes with NF = 50 (composed by one pair of two-armed chromosomes – m, sm, or st) plus 46 acrocentric elements) are shared by 35% of the species and constitute the most widespread condition among Carangidae tribes (Chai et al. 2009; present data). However, this probable basal constitution for the family shows increasing evolutionary divergences among tribes mainly modeled by pericentric inversions (Sola et al. 1997; Rodrigues et al. 2007). Other chromosome rearragements, such as centric fissions and fusions, have a lesser extent on the karyotype differentiation. In fact, fissions are phylogenetically restricted and detected only in three Carangini species, leading to the increase from the basal 2n = 48 to 2n = 50 and 2n = 56 (Table 1). Likewise, Robertsonian fusions are also rare events, with a polymorphic pattern in *Serioladumerili* (Risso, 1810) (2n = 48/47) (Vitturi et al. 1986) and a stable condition in *Selenesetapinnis* (Mitchill, 1815) (2n = 46) (Jacobina et al. 2013).

Pericentric inversions are predominant changes in the order, but to a lesser extent in the syntenic composition of gene groups. If so, the chromosome conservation evidenced among the four Carangidae genera could encompass a wide shared synteny among the species. Indeed, genetic maps of *Seriola* species evidenced a high collinearity among their linkage groups (Ohara et al. 2005), thus supporting this hypothesis. Additionally, comparison of carangid genome assemblies (Zhang et al. 2019), including *Trachinotusovatus* (Linnaeus, 1758) (2m+4sm+42st/a; Choudhury et al. 1993), *Seriolaquinqueradiata* Temminck et Schlegel, 1845 (2sm+2st+44a; Ida et al. 1978), *S.dumerili* (2sm+2st+44a; Sola et al. 1997), and *S.rivoliana* Valenciennes, 1833 (2sm+2st+44a; present data) revealed synteny with *T.ovatus* 24 linkage groups, indicating that fission and/or fusion events are unlikely during their karyotype evolution.

If pericentric inversions are the most common rearrangements in Carangidae, and if they are equally likely to occur in all tribes, a similar level of karyotype divergence among them would be expected. However, this does not occur, as seen by cytogenetic data covering representative species from most genera of each tribe (Table 1). Since tribes share a common origin, what factors would be linked with this differential fixation of chromosome rearrangements? Data show that, on average, species of the Naucratinae tribe has NF = 51.7, the closest one to that considered basal (NF = 50) for the family, followed by species from the Scomberoidini (NF = 52), Caranginae (NF = 53.4), and Trachinotini (NF = 54.4) tribes. Notably, the level of karyotype diversification of these groups was inversely proportional to their geographic distribution, thus suggesting that the dispersive potential and, consequently, the level of gene flow maintained by migrants) are agents driving the karyotype evolution in the group.

The geographic variables MLD and OA showed a high positive correlation with each other and both showed a negative correlation with the NF (*p* ≤ 0.05). In fact, the data set revealed that the probability of chromosomal variations decreases as the geographical distribution of the species expands. Between the two distribution variables, MLD exhibited a more pronounced negative correlation with the NF. Although both parameters are negatively associated with chromosomal variation, they have different prediction intervals. The modest correlation between OA and NF, was statistically significant, and probably related to lower precision in the definition of the ecological areas occupied by the species. In contrast, MLD, despite being a simpler parameter, proved to be a more effective predictor of differences in the dispersive potential of migratory species.

**Figure 2.**
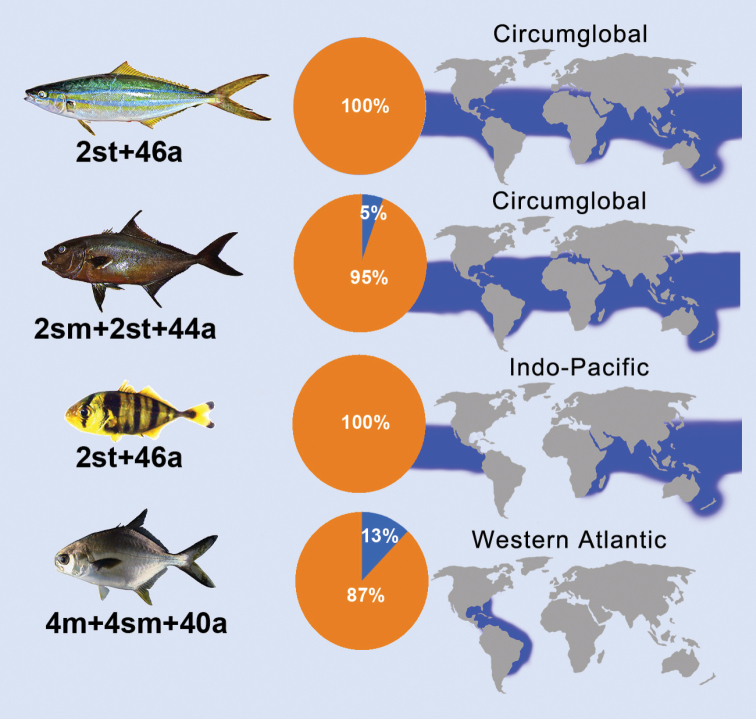
Karyotype index of chromosomal similarity (orange) and divergence (blue) regarding the probable basal karyotype for Carangidae studied species. Maps show the magnitude of the geographic distribution of *Elagatisbipinnulata*, *Seriolarivoliana*, *Gnathanodonspeciosus*, and *Trachinotuscarolinus* (top to bottom).

Large pelagic fish populations, whose life histories include migratory behavior, planktonic larval stages, and broadcast spawning, maintain high levels of gene flow among vast oceanic areas (Pla and Pujolar 1999; Tripp-Valdez et al. 2010), thus finding fewer opportunities for fixing chromosome rearrangements and, essentially, maintaining a more conservative karyotype evolution (Molina 2007; Accioly et al. 2012; Soares et al. 2013; Molina et al. 2014; Motta-Neto et al. 2019). Having that in mind, the low rate of NF divergence in Naucratini is probably due to the wide distribution of some *Elagatis*, *Seriola*, and *Seriolina* Wakiya, 1924 species reaching circumglobal scales (Froese and Pauly 2020). In contrast, Carangini, the most diverse Carangidae tribe, shows the largest ranges in the diploid number and NF, from 46 to 56 and 48 to 78, respectively (Table 1). In this group, several species have a circumglobal distribution (e.g., *Gnathanodonspeciosus* and *Caranxlugubris* Poey, 1860), which show the basal karyotype pattern for the family. In spite of this group has an average NF conspicuously higher than that of Naucratini, this value is strongly biased by *Trachurus* Rafinesque, 1810, species, which have higher NF values (NF = 50–78). In fact, *Trachurus* diverge markedly from other Carangini groups because its species have a limited distribution (FAO 2020), with evidence of strong genetic structuring between broad and distant regions (Karaiskou et al. 2004). Thus, the analysis of the structural diversification of Carangini karyotypes, removing the particular group *Trachurus* (Fig. 2), drastically reduces the NF values for this tribe, making the NF = 51.3, thus very close to that of the Naucratini subfamily. On the other hand, Trachinotini species showed the lowest geographic distributions among the other tribes and the more divergent NF values.

Significantly, a conservatism pattern can also be seen at microstructural cytogenetic level. For example, the 18S rDNA sites, that are usually characterized by a high evolutionary dynamism among fishes (Gornung 2013), have a stable distribution pattern among the four species now analyzed, as well as in several other Carangidae species (Accioly et al. 2012; Jacobina et al. 2012, 2014a). Their similarity in number and chromosomal location probably represent a homeolog linkage group among them. Although the 5S rDNA sites exhibit a more dynamic evolutionary pattern in other species (Accioly et al. 2012; Jacobina et al. 2012, 2013, 2014a, b), they also reveal here signs of microstructural conservatism.

The distribution of some *Trachinotus* species in the Western Atlantic is subdivided by the Amazonas and Orinoco rivers barrier. In this context, *T.carolinus* from Caribbean, first analyzed here, shows no variable karyotypes compared to those previously reported for populations from the southeast and northeast Brazilian coasts (Rodrigues et al. 2007; Jacobina et al. 2012).

Biogeographic barriers in marine oceans affect the karyotype diversification (Molina et al. 2012), but have different effects among the species (Motta-Neto et al. 2019). In this context, cytogenetic analyses in fish populations from different biogeographic regions help to decipher the karyotype evolution in groups with large distribution.

## ﻿Conclusions

Carangidae constitute a marine fish group in which many species are vagrant/nomadic pelagic swimmers, ranging from a single ocean to circumglobal distributions. Gene flow among marine fish populations with significant population sizes and extensive distributions can mitigate genetic differentiation. The cytogenetical/geographical approach suggest negative correlation between active migratory capacity and cytogenetic divergence in marine fish. This genetic context could restrains evolutionary diversification and speciation, in the Carangidae, a clade in which many genera are monotypic or formed by a few species. As a whole, our data provide preliminary data of high gene flow in minimize chromosomal rearrangements in large oceanic spaces, highlighting new scenarios of the karyotype evolution in pelagic species.

## ﻿Authors’ contributions

**Rodrigo Xavier Soares**: Conceptualization, Methodology, Writing – Original draft preparation, Data curation. **Gideão Wagner Werneck Félix da Costa**: Investigation, Validation. **Clóvis Coutinho da Motta-Neto, Amanda Torres Borges**: Supervision, Visualization. **Marcelo de Bello Cioffi, Luiz Antônio Carlos Bertollo**: Writing – Reviewing and Editing. **Wagner Franco Molina**: Conceptualization, Methodology, Writing – Original draft preparation, Funding acquisition, Project administration. Writing – Reviewing and Editing.

## ﻿Data availability statement

The data that support the findings of this study are available from the corresponding author upon reasonable request.
